# *Mitragyna speciosa* Korth toxicity: Experimental findings and future prospects

**DOI:** 10.1016/j.jtumed.2024.12.002

**Published:** 2024-12-12

**Authors:** Taslima Begum, Mohd H. Arzmi, A.B.M. Helal Uddin, Alfi Khatib, Syed A. Abbas, Qamar U. Ahmed

**Affiliations:** a Drug Discovery and Synthetic Chemistry Research Group, Department of Pharmaceutical Chemistry, Kulliyyah of Pharmacy, International Islamic University Malaysia, Kuantan, Pahang, Malaysia; b Department of Fundamental Dental and Medical Sciences, Kulliyyah of Dentistry, International Islamic University Malaysia, Kuantan, Pahang, Malaysia; c Cluster of Cancer Research Initiative IIUM, International Islamic University Malaysia, Kuantan, Pahang, Malaysia; d Melbourne Dental School, The University of Melbourne, Swanston Street, Victoria, Australia; e Department of Pharmacology, Faculty of Pharmacy, Quest International University, Ipoh, Perak, Malaysia

**Keywords:** القرطوم, ميتراجينا سبيسيوزا, الميتراجينين, السمية, سمية الكبد, سمية القلب, Cardiotoxicity, Hepatotoxicity, Kratom, *Mitragyna speciosa*, Mitragynine, Toxicity

## Abstract

*Mitragyna speciosa* (Roxb.) Korth, locally known as kratom, is a traditional medicinal plant from Southeast Asia, with mitragynine as its principal alkaloid. Similar to other medicinal plants, kratom has side effects and toxicities, which have been documented in scientific studies and case reports. The mitragynine sale and possession of kratom are prohibited in Malaysia but legalized in Thailand. In the US, kratom is not lawfully marketed as a drug product, a dietary supplement, or a food additive in conventional food. Despite these restrictions, individuals continue to self-administer kratom to alleviate various health problems, often without a comprehensive understanding of the associated toxicities. Hence, the primary aim of this review is to provide a comprehensive overview of the toxicities associated with kratom, drawing from scientific studies, case reports, and other relevant sources. It also addresses the management of these toxicities, identifies gaps in existing studies, and discusses future perspectives. Therefore, a literature review search was conducted to gather essential information for this review. The *in vitro* studies focused on metabolizing enzymes, indirectly indicating kratom toxicity. By contrast, the *in vivo* results directly demonstrated kratom's toxic effects on the liver, kidneys, lungs, and brain. Case studies, primarily from Western countries, involved both single and combination use of kratom. Thus, by shedding light on these aspects, we aim to enhance awareness among healthcare professionals and the general public. Additionally, identifying existing gaps can guide future scientific studies. Since prevention is better than cure, this review holistically presents information about the toxicities associated with the use of kratom leaves, serving anyone seeking to understand and prevent kratom-related toxicities.

## الملخص

**أهداف البحث:**

نبات القرطوم، المعروف علميا باسم ميتراجينا سبيسيوزا، هو نبات طبي تقليدي من جنوب شرق آسيا، يحتوي على قلويد الميتراجينين كمركب رئيسي. ومثل النباتات الطبية الأخرى، له آثار جانبية وتأثيرات سمية موثقة في الدراسات العلمية وتقارير الحالات. يحظر بيع وحيازة القرطوم في ماليزيا لكنه قانوني في تايلاند. في الولايات المتحدة، لا يسوق القرطوم قانونيا كمنتج دوائي أو مكمل غذائي أو مضاف للأغذية التقليدية. رغم هذه القيود، يستمر الأفراد في تعاطي القرطوم ذاتيا لتخفيف مشاكل صحية متنوعة، غالبا دون فهم شامل للسميات المرتبطة به.

**طرق البحث:**

تم إجراء بحث مرجعي لجمع المعلومات الأساسية لهذه المراجعة. ركزت الدراسات المخبرية على إنزيمات الأيض، مشيرة بشكل غير مباشر إلى سمية القرطوم. في المقابل، أظهرت نتائج الدراسات على الحيوانات التأثيرات السمية المباشرة للقرطوم على الكبد والكلى والرئتين والدماغ. تضمنت دراسات الحالة، التي أجريت بشكل رئيسي في الدول الغربية، استخدام القرطوم منفردا أو مع مواد أخرى.

**النتائج:**

تشير الدراسات إلى وجود تأثيرات سمية متعددة للقرطوم على أعضاء الجسم المختلفة. وتؤكد دراسات الحالة هذه المخاطر من خلال توثيق حالات التسمم المختلفة.

**الاستنتاجات:**

تهدف هذه المراجعة إلى تعزيز الوعي بين المتخصصين في الرعاية الصحية وعامة الناس حول مخاطر القرطوم. كما يساعد تحديد الفجوات الحالية في توجيه الدراسات العلمية المستقبلية. وبما أن الوقاية خير من العلاج، تقدم هذه المراجعة معلومات شاملة عن السميات المرتبطة باستخدام أوراق القرطوم.

## Introduction

**Figure 1:**
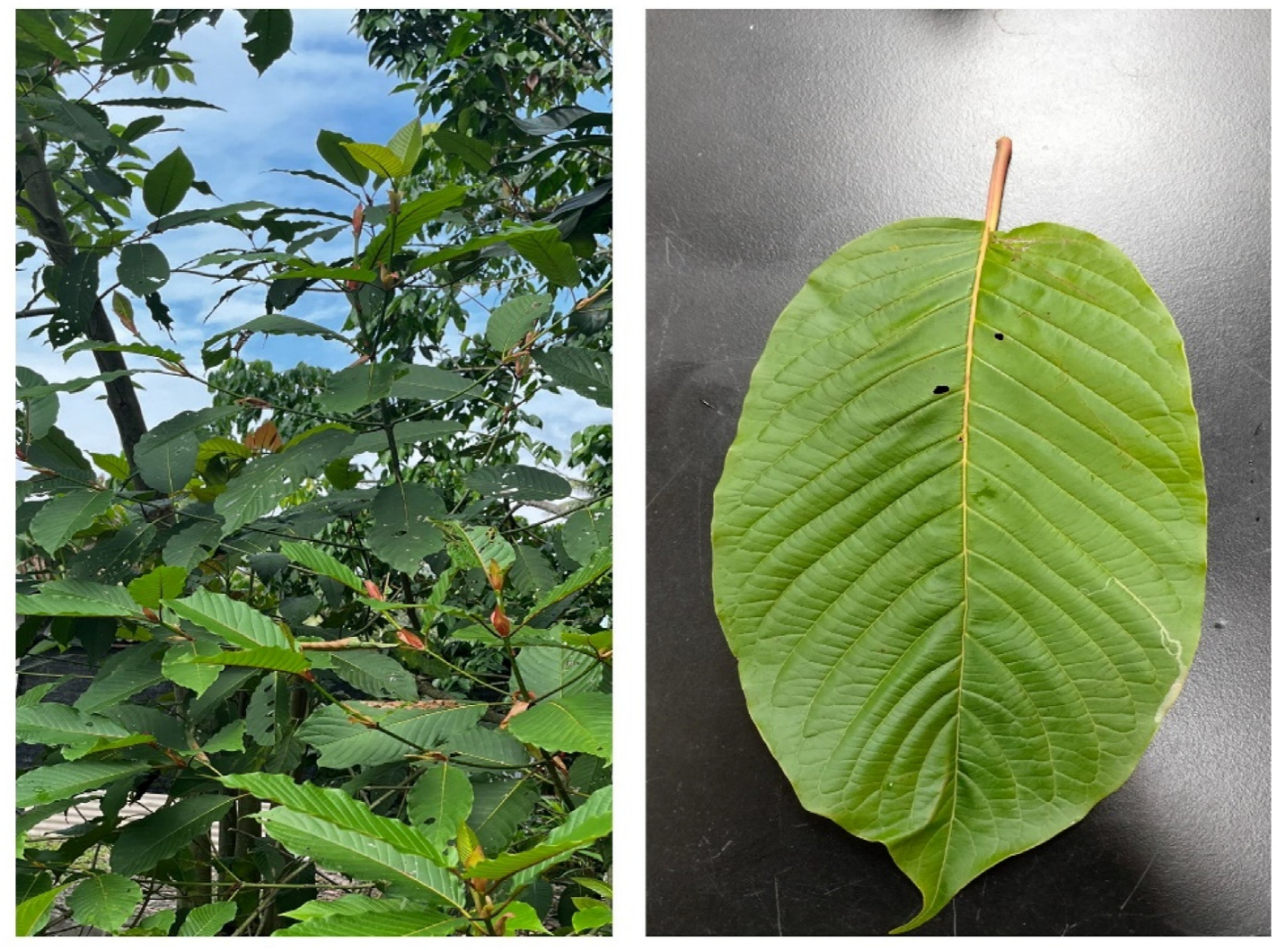
Kratom leaves.

*Mitragyna speciosa* (Roxb.) Korth, locally known as kratom, ketum, or biak–biak in Malaysia, is a native plant of Southeast Asia, with large, oval-shaped leaves of dark green color (Figure 1). The leaves of this plant are usually 18 cm in length and 10 cm in width, whereas the flowers are of a deep yellow color.^1^^,^^2^ The local population utilizes the leaves to treat various health problems, including pain, fever, cough, anxiety, hypertension, diabetes, opioid withdrawal symptoms, diarrhea, and intestinal infection. Additionally, the leaves are used to prevent cancer and enhance sexual desire.^2^, ^3^, ^4^, ^5^, ^6^, ^7^

In Southeast Asia, kratom leaves are administered in several ways to achieve the desired biological effects. For instance, the fresh leaves can be chewed, smoked, infused into herbal tea, or mixed with coffee or liquid refreshments.^8^^,^^9^ Moreover, recently in Thailand, the younger generation is using the drug as a cocktail drink called ‘4x100’, which contains kratom tea (base), Coca-Cola, cough syrup along with assorted items (analgesics, antidepressants) based on personal preferences.^10^^,^^11^ In recent years, increased commercial kratom preparation has made it available to regions far from its origins. There is evidence indicating that kratom consumption is increasing in the US,^12^ Europe,^13^ and Japan.^14^ In Western countries, *M. speciosa* leaves are available in various forms, including dried leaves for chewing or brewing, as well as tablets, capsules, and for smoking.^1^^,^^15^

Due to the wide availability of kratom leaves, the number of toxicity incidences is also rising. Apart from the beneficial effects, tolerance and withdrawal symptoms have been reported as negative psychosocial impacts.^16^ Chronic use of the leaves has been reported to cause darkening of the skin, constipation, frequent urination, insomnia, dehydration, weight loss, tiredness, and lack of sexual desire.^6^^,^^9^, ^17^ In Thailand, kratom constitutes about 2 % of admission for drug-related treatment.^18^ There are reports that *M. speciosa* can exert toxicity in humans. A review on the kratom toxicity in the US found that the National Poison Data System reported 2312 kratom exposures. Of these, 935 cases involved single kratom exposure. Several adverse drug reactions such as agitation (18.6 %), tachycardia (16.9 %), and vomiting (11.2 %) were noted. Severe adverse effects included respiratory depression (2.8 %), coma (2.3 %), and cardiac or respiratory arrest (0.6 %).^19^

Scientific studies, especially *in vitro* studies, have proven that kratom can interfere with the metabolism of other drugs when taken concomitantly. This interaction is also evident in case studies, where most reported toxicities occurred when kratom was taken with other drugs. Additionally, case studies have observed the toxicity incidences even if the individuals stopped consuming alcohol 2 years prior to starting kratom consumption.^20^ This indicates that the physiological changes resulting from chronic alcohol consumption may affect the body, potentially leading to toxicity when kratom is taken. Additionally, several case studies noted that subjects had previous health complications such as surgery, cancer, hypertension, and hyperlipidemia.^21^^,^^22^ Conversely, *in vivo* results revealed potent toxic effects of kratom on various organ systems. Therefore, this study incorporated all toxicities associated with the use of kratom, whether taken alone or in combination, to provide a comprehensive overview of kratom-related toxicity.

**Figure 2:**
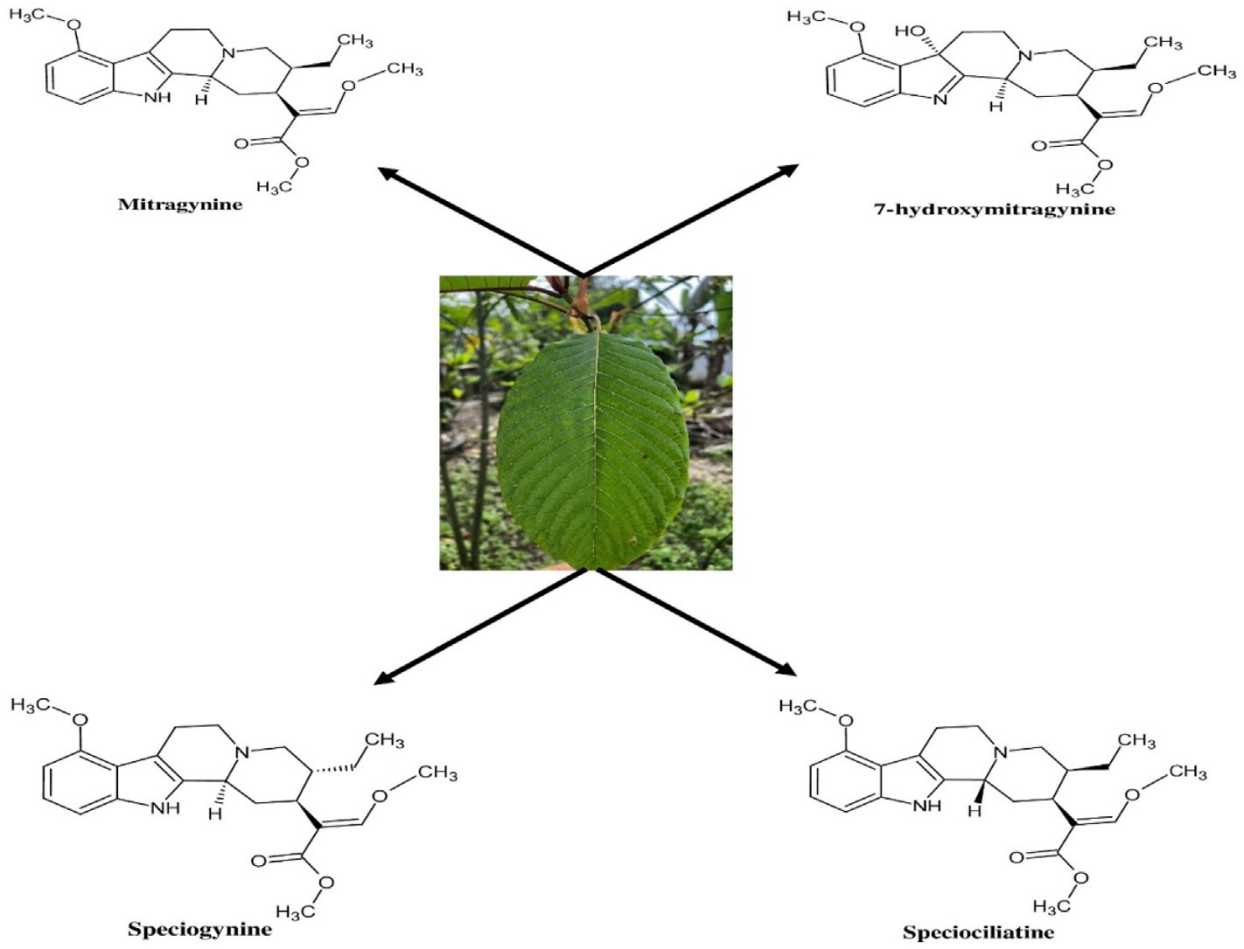
Kratom leaf and chemical structures of some alkaloids obtained from it.^25^

Kratom leaves contain a mixture of alkaloids and other phytochemical compounds such as saponins, terpenoids, triterpenoids, polyphenols, and flavonoids.^23^ Generally, alkaloids with pyrrolizidine, piperidine, tropane, and indolizidine in their chemical structures reportedly exhibit toxicity in humans and animals.^24^ Therefore, the chemical structures of the alkaloids present in kratom themselves provide a strong baseline for kratom toxicity due to the occurrence of the piperidine ring in their chemical structures.^16^ The chemical structures of some most pronounced kratom alkaloids are shown in Figure 2.^25^

**Figure 3:**
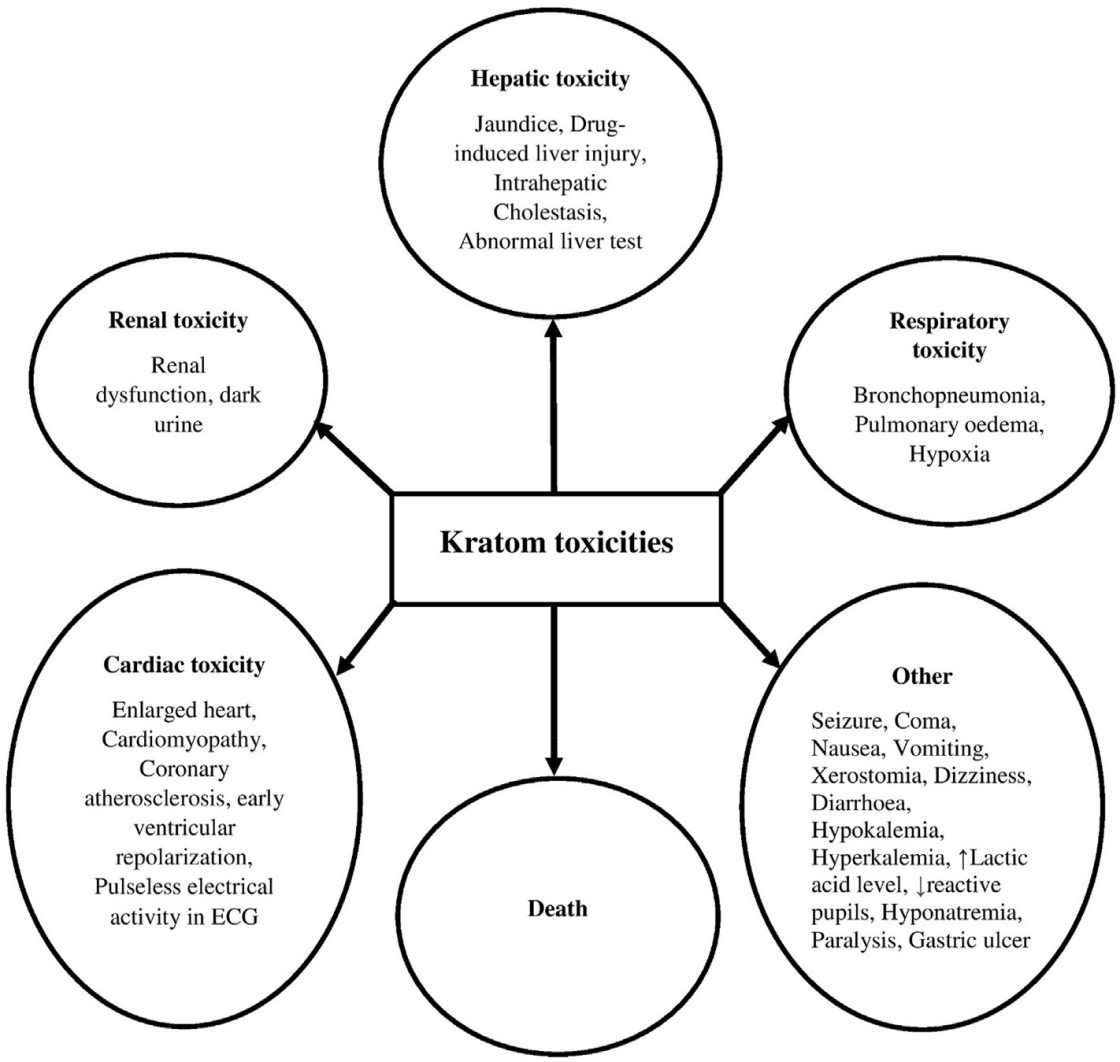
Toxicities associated with use of kratom, obtained from case studies.^20^, ^21^, ^22^^,^^45^, ^46^, ^47^, ^48^, ^49^, ^50^, ^51^, ^52^, ^53^, ^54^, ^55^, ^56^, ^57^, ^58^, ^59^, ^60^, ^61^, ^62^, ^63^, ^64^, ^65^

This article aims to compile data on the toxicities associated with kratom leaf administration, including findings from scientific studies (both *in vitro* and *in vivo*), case studies, and surveys. The main goal is to provide a comprehensive resource on kratom toxicity for anyone seeking information about the adverse effects of kratom use and their management. Additionally, the gaps in the existing studies have been identified to highlight areas for future improvement.

## Materials and Methods

The qualifying criteria for this review were established by examining relevant articles. The irrelevant data were carefully filtered out from the articles, titles, and abstracts of academic papers. Studies were chosen based on the subsequent inclusion criteria: (1) articles published in English; (2) articles discussing the traditional uses of kratom, its toxicity, *in vitro* and *in vivo* toxicity, and case studies on kratom toxicity; (3) articles with full-text access; (4) papers reporting toxicities from kratom alone or in combination with other drugs; (5) no publishing date restrictions, allowing for the inclusion of scientific papers from 1930 to 2024 to gather comprehensive information on kratom toxicity and management; and (6) information sourced from PubMed, Science Direct, ProQuest, Google Scholar, and Web of Science. The exclusion criteria were as follows: (1) articles published in non-English languages; (2) studies with uncertain methods, data, and outcomes; and (3) letters, conference papers, posters, editorials, and studies published in disputed or predatory journals.

The quality of the research articles included in this review was independently evaluated by all co-authors using several key criteria. First, the relevance of each article to the topic of interest was considered. Additionally, the reputation of the journal in which the article was published was considered. Furthermore, the methodology was scrutinized to ensure that the research design was clearly assessed, and that the data collection and analysis methods were appropriate and rigorously applied. It was also determined whether the methodology was clearly described and cited with genuine reference.

## Results

### Toxicity of kratom

Kratom toxicity is not a new phenomenon although it has become more pronounced recently due to the increased number of toxicity cases. One of the early documentations of the side effects and toxicities associated with kratom exposure was outlined by Suwanlert,^4^ who reported that chronic administration of kratom is associated with loss of appetite, weight loss, insomnia, darkening of the skin, and xerostomia (dry mouth). Additionally, kratom withdrawal symptoms include muscle pain and bone pain, restlessness, hostility and decreased workability.

Currently, kratom toxicity has become a significant concern due to the commercialization of kratom products from its origin country to Western countries. A wide range of side effects and toxicities have been encountered and recorded by the toxicity management units. Consequently, numerous scientific studies have been conducted on kratom extracts and isolated alkaloids, including both *in vitro* and *in vivo* studies, to evaluate their toxic potential. Along with this, various case studies and surveys are also available to shed light on the side effects and toxicities associated with kratom exposure.

### *In vitro* toxicity studies of kratom

Several *in vitro* studies have been performed on different extracts as well as alkaloids obtained from this plant to elucidate the toxic effects of kratom leaves. Generally, most drugs are considered xenobiotics, which undergo chemical alterations in the body to be excreted easily. This process, known as drug metabolism, primarily occurs in the liver, which contains a range of metabolizing enzymes.^26^ These enzymes include cytochrome P450s (CYPs), UDP-glucuronyl transferases (UGTs), sulfotransferases such as glutathione S-transferase and esterase such as carboxylesterase 1 (CES1).^27^^,^^28^ Any effect on these enzymes can alter the metabolism, leading to drug interactions and serious toxicities. Therefore, the *in vitro* toxicity studies of kratom and its alkaloids were targeted at various liver enzymes.

Among them, one study evaluated the impacts of alkaloid extract of kratom on recombinant CYP enzymes using the modified Crespi method. The outcomes evidenced that against CYP3A4 and CYP2D6, the alkaloid extract had inhibitory activity with half-maximal inhibitory concentration (IC_50_) values of 0.78 and 0.636 μg/mL, respectively. The extract showed moderate and weak inhibition against CYP1A2 and CYP2C19, respectively. Therefore, this study concluded that kratom extract can initiate kratom–drug interactions when co-administered with drugs metabolized by CYP3A4, CYP2D6, and CYP1A2 enzymes.^29^

Additionally, another study investigated the inhibitory potential of three kratom extracts with different origins (denoted as extract A, B, and C) and alkaloids, namely mitragynine, speciogynine, speciociliatine, paynantheine, and corynantheidine on the liver hydrolase enzyme CES1. The results revealed that among the three extracts, extract A exhibited the highest CES1 inhibition, and had comparatively high levels of mitragynine, paynantheine, speciociliatine, speciogynine, and corynantheidine. Although all of the test compounds showed dose-dependent reversible inhibition of CES1, mitragynine showed full competitive inhibition, whereas speciociliatine, paynantheine, and corynantheidine elicited mixed-type reversible inhibitory effects.^27^

Additionally, Azizi et al.^28^ evaluated the impacts of methanol, aqueous, and alkaloid-rich extracts (0.01–750 μg/mL) of *M. speciosa* on glutathione transferase-specific activity. The outcome showed that all of the extracts elicited dose-dependent enzyme inhibitory activity. At high doses, methanol showed the most potent inhibitory effect (61 %), followed by the aqueous and total alkaloid extracts (50 % and 43 %, respectively). Another *in vitro* study assessed methanol, aqueous, and alkaloid extracts on aminopyrine N-demethylase and UGT using rat liver cytosolic and microsomal fractions. The study revealed that the methanol extract most effectively inhibited the activity of both enzymes, followed by the aqueous and alkaloid extracts.^30^

The cytotoxic effects of mitragynine were determined by a scientific study on the SH-SY5Y human neuronal cell line. It was observed that mitragynine-treated cells exhibited a dose-dependent reduction in colony-forming ability, thereby reducing cell survival. Further studies with metabolically active MCL-5 cells and chemical inhibitors revealed that the cytotoxicity was related to CYP2E1 and CYP2A6 enzymes. However, outcomes of the L5178 TK ± mouse lymphoma assay showed that mitragynine was not genotoxic. Additionally, the cytotoxic effects of mitragynine were partially antagonized by naloxone. This indicates that the cytotoxic effects of mitragynine are mediated partly via opioid pathways, similar to familiar opioid toxicities. Finally, the study concluded that high doses of mitragynine can pose a risk to heavy alcohol consumers due to their high CYP2E1 activity.^31^ Conversely, another study observed that mitragynine (1–25 μM) generated dose-dependent elevation in both mRNA and protein expression along with activity of CYP1A2, CYP2D6, and CYP3A4 enzymes in human liver cells.^32^

Beside these, one study evaluated the potential of kratom leaves to produce drug interactions by assessing the receptor binding capacity of speciofoline, mitragynine, and 7-hydroxymitragynine present in the leaves of kratom. The findings revealed that mitragynine and 7-hydroxymitragynine acted as partial agonists of the human μ-opioid receptor. By contrast, speciofoline does not have binding affinity against any of the opioid receptors. Additionally, mitragynine and 7-hydroxymitragynine presented functional selectivity for G-protein signaling. All of the tested alkaloids inhibited several CYP450 enzymes, posing a significant risk of drug interactions. Therefore, when kratom leaves are taken with drugs metabolized by these enzymes, they can increase the systemic availability of the drugs, thereby exerting toxic effects.^33^

Another study also evaluated the potential of kratom alkaloids, mitragynine, 7-hydroxymitragynine, speciogynine, speciocilliatine, corynantheidine, and paynantheine, on human liver microsomes to produce drug–drug interactions. The results showed that mitragynine exhibited selective inhibition of CYP2D6 (with an IC_50_ of 2.2 μM) and moderate inhibition of CYP3A4/5 (with an IC_50_ of 11.4 μM). However, no significant activity was observed for mitragynine, speciogynine, and speciociliatine towards other CYP450 isoforms. Hence, this study concluded that kratom alkaloids can potentiate drug interactions by blocking CYP450 isomers.^34^ Conversely, three kratom alkaloids, namely, mitragynine, speciogynine, and speciociliatine, were studied for their cardiotoxicity in human-induced pluripotent stem cell-derived cardiomyocytes. The outcomes revealed that mitragynine (10 μM) blocked the current of human ether-à-go-go-related genes, increased action potential direction, and produced arrhythmia. Finally, it was suggested that all of these alkaloids might produce Torsade de Pointes via inhibition of potassium current in human cardiomyocytes.^35^

Therefore, the aforementioned studies revealed the impacts of kratom and its alkaloids on various liver enzymes. Thus, co-administration of kratom and drugs metabolized by these liver enzymes can create kratom–drug interactions, which may prove deleterious. Some of these drugs are warfarin (CYP2C9), benzodiazepines (CYP3A), and desipramine and dextromethorphan (CYP2D6).^36^ Additionally, some well-known drugs metabolized by CES1 include clopidogrel, oseltamivir, and methylphenidate.^27^ Hence, from all of the aforementioned studies on various metabolizing enzymes, it is noteworthy that kratom itself was not directly responsible for toxicity. Instead, by increasing the systemic availability of concomitantly administered drugs, which were substrates for these enzymes, the toxicities are actually generated.

### *In vivo* toxicity studies of kratom

The *in vivo* studies on kratom toxicity were performed mainly on *Sprague Dawley (SD)* rats to assess acute and subchronic toxicity.^37^, ^38^, ^39^, ^40^ Moreover, zebrafish embryo and brine shrimp were used as animal models to evaluate gestational toxicity and lethality, respectively.^41^^,^^42^ The *in vivo* toxicity test results exhibited outcomes similar to those observed in the *in vitro* results; no direct lethality was observed. In most studies, biochemical parameters of the hepatic system (alanine transaminase [ALT], aspartate transaminase [AST], bilirubin, albumin) and renal system (urea, creatinine, gamma-glutamyl transferase [GGT]) were increased after treatment. Apart from the elevated biochemical parameters, some other signs of toxicity have been reported by several studies. Among them, Harizal et al.^38^ performed an acute toxicity study with methanol extract and observed that at a dose of 1000 mg/kg body weight, significant hepatic damage was induced including congestion of sinusoids, hemorrhage, lipid accumulation, and centrilobular necrosis. These effects were similar to those of morphine. However, the kidney, brain, and lung showed no noteworthy morphological changes. Similarly, Ilmie et al.^39^ reported no histopathological changes in the brain, spinal cord, and heart upon subchronic exposure to methanol extract. Unlike the previously mentioned study, significant toxicity was observed in the liver, lung, and kidney at doses of 200 and 500 mg/kg.

Another subchronic toxicity study^43^ observed no toxicity in the lung, heart, and spleen. However, this study identified toxicity in the nervous system, as well as in the hepatic system (liver cell hypertrophy, dilation of sinusoids, hemorrhage in liver cells) and renal system (swollen glomerulus capsule and the presence of red blood cells between lumens). At a dose of 100 mg/kg/body weight, the extract caused damage to the medulla base via local vacuolation and necrosis of neuronal cells. Similar toxicities were also observed in the hippocampus, frontal lobe, and cerebellum by histopathologic analysis. Thus, it has been noted from the *in vivo* studies that kratom extracts, whether in acute or subchronic conditions, induce hepatic toxicity, with the intensity of toxicity increasing in the latter condition. Table 1 illustrates *in vivo* kratom toxicity in various animal models.

**Table 1:** *In vivo* toxicity studies of kratom extracts and alkaloids.

Extracts/Compounds/Mode of delivery	Experimental model/Test	Results/Conclusions	References
Aqueous ext. (175, 500, and 2000 mg/kg/bw), oral	Male & female SD rats of age 8 and 12 weeks old, acute toxicity.	No notable variations were observed in case of bw and internal organ weight, as well as food and water ingestion. However, reduction in hemoglobin concentration, albumin, calcium, and cholesterol were observed. Additionally, several segments of the liver got steatosis and centrilobular necrosis. Therefore, the extract generated little toxicity without creating any death.	37
MeOH ext. (100, 500, and 1000 mg/kg/bw), oral	Albino SD rats of 4 weeks old with weight of 150–200 g, acute toxicity.	No incidence of mortality was detected along with no change in behavior, organ weights, food and water intake, and hematological tests, although an elevation of blood pressure occurred after 1 h of administration. Additionally, the extract produced acute severe liver toxicity (AST [U/L] 125.17 ± 3.60, albumin (Abs) 39.33 ± 0.38, ALT (U/L) 73.10 ± 2.79) and mild nephron toxicity (urea [mmol/L] 6.64 ± 0.20, creatinine 44.10 ± 0.93, GGT [U/L] 16.64 ± 0.50) at high doses.	38
MeOH ext. (100, 200, and 500 mg/kg/bw), oral for 28 days	Male SD rat (50–60 g and 4 weeks), subchronic toxic effect.	The extract had significant effects on body weight and elevated values were obtained for albumin, ALT, creatinine, globulin, glucose, urea, and total protein. However, AST values decreased with increasing the dose of the extract. Hence, the hepatic and renal systems were affected. Compared to other organs, the hepatic, renal, and pulmonary systems were affected more. Therefore, this study evidenced subchronic toxicity of MeOH extract.	39
Kratom decoction (either vehicle or extract with doses 10, 50, and 150 mg/kg/bw; 2 groups were given both vehicle and decoction with a dose of 150 mg/kg/bw), oral	Male and female SD rats, safety and toxicity profile after 28 days of treatment.	The results indicated no incidence of death and no changes in bw or hematological parameters, except a reduction in platelet count. Biochemical tests revealed elevated levels of AST, ALT, uric acid, and alkaline phosphatase. Conversely, histological tests detected damage to liver, kidney, and brain tissues, with no changes observed in the heart and lungs. Thus, this study demonstrated hepatic and renal toxicity with multiple doses, without any evidence of death.	40
Kratom leaves decoction (1000–62.5 μg/mL), mitragynine and speciociliatine (100–3.125 μg/mL)	Zebrafish embryo, mortality, hatching rate, heart rate, and morphological abnormalities were assessed at 24, 48, 72, and 96 hpf, respectively.	Kratom decoction at ≥500 μg/mL dose generated 100 % mortality and the hatching rate was reduced by following dose dependent manner. 100 % mortality of embryos was observed for mitragynine and speciociliatine at a dose of 100 μg/mL. Moreover, at high doses, both of these alkaloids significantly inhibited hatching and caused scoliosis. Thus, at high doses, kratom decoction along with its two alkaloids generated gestational toxicity, thereby giving warrants for probable teratogenic toxicity during pregnancy.	41
Mitragynine, alkaloid ext. and aqueous ext. (10–100 μL/mL)	Brine shrimp, brine shrimp lethality assay.	LC_50_ of mitragynine, alkaloid ext. And aqueous ext. Were 44, 62, and 98 μL/mL, respectively. Therefore, among the three test compounds, mitragynine was proved to be more toxic.	42
Mitragynine (1, 10, and 100 mg/kg/bw), oral for 28 days	Male and female SD rat (5 weeks old), subchronic toxicity.	Overall, all treatment groups showed no incidence of lethality and lower and intermediate doses exhibited no toxic effects. High doses produced less food consumption and weight reduction only in female rats. However, both male and female animals treated with the high dose showed an increase in liver weight and alteration in biochemical and hematological test results. Therefore, mitragynine at a high dose showed toxicity in subchronic conditions.	43

### Case studies on kratom toxicity

The aforementioned *in vitro* and *in vivo* study results aligned with the outcomes of various case studies, which report hepatic, renal, and cardiac complications. According to *in vitro* toxicity studies, kratom can inhibit liver enzymes, thereby posing a significant risk of drug–drug interactions. Most case studies involve the concomitant use of other drugs (either conventional or illicit) with kratom. Hence, it cannot be exclusively claimed that the toxicities observed in these case studies are solely due to kratom; they might also result from high blood concentrations of other drugs used. Conversely, *in vivo* toxicity results provide evidence of toxicities caused by kratom on various body systems. Thus, we cannot nullify the notion that the toxicities reported in the case studies were also due to kratom. Consequently, this review considers all case studies reporting toxicities associated with kratom use, whether alone or in combination with other drugs, as kratom toxicities.

The case studies present an exception to the *in vitro* and *in vivo* results, which is death. Although the scientific studies evidenced no direct lethality, several case studies report deaths associated with kratom intake. Researchers believe that the combination of kratom with either illegal or conventional drugs might be responsible deaths in Western countries.^44^ Apart from these, the case studies also reported kratom toxicities in the forms of symptoms similar to opioid withdrawal (nausea, vomiting, diarrhea) as well as xerostomia and short time paralysis. Therefore, this review has divided the toxicities associated with kratom use, obtained from case studies into four separate tables to facilitate the discussion. Table 2 contains case studies on toxicities associated with kratom use on hepatic, renal, pulmonary, and cardiac systems. Table 3 contains case studies on toxicities associated with kratom use similar to opioid withdrawal symptoms. Table 4 contains case studies on death associated with kratom use and Table 5 contains case studies on toxicities associated with kratom use other than the above. Additionally, Figure 3 comprehensively illustrates all toxicities associated with the use of kratom, obtained from case studies.

**Table 2:** Case studies on toxicities associated with kratom use on hepatic, renal, pulmonary, and cardiac systems.

Age and sex	Dose & mode of administration	Kratom toxicity	References
23-year-old man	Kratom was taken at a dose of 30 g per day for 14 days within the previous month and the last dose was just 1 week before the symptoms commenced. He used to take vapes and smokes marijuana daily.	Jaundice, diffuse itching, pale stools, dark urine, abdominal discomfort, and excessive fatigue.	45
47-year-old man	The subject was a heavy alcohol consumer over 1 year, although he stopped consuming alcohol 2 years ago. Kratom was taken 3 weeks before symptom presentation.	Jaundice for the past 5 days. Urine drug analysis revealed the presence of tetrahydrocannabinol and benzodiazepines.	20
23-year-old man	The subject used to take opioids and kratom. On symptom presentation day, he took beer. Moreover, the patient tested positive for COVID-19.	Cardiac arrest with no breathing, ECG had pulseless electrical activity, and tested positive with COVID-19. Also diagnosed with early ventricular repolarization syndrome.	46
47-year-old man	The subject started taking kratom capsule 3 weeks before symptom presentation and took it several times but not on a daily basis with a view of managing his lower back pain. Apart from this, to treat mild fevers along with other symptoms over 2 days before presentation, he took acetaminophen up to 3000 mg/day. His current medications were clonazepam, fexofenadine valsartan, metoprolol tartrate, and escitalopram.	Fatigue, pruritus, and abnormal liver tests, characterized as drug-induced liver injury (DILI).	21
45-year-old woman	Administration of kratom was 1 month previously and gradually increased the dose. The subject had a prior history of appendectomy, hysterectomy, ureteral stents, surgery for bowel obstruction, and breast reconstruction surgery etc.	Rhabdomyolysis, compartment syndrome, hepatic and renal dysfunction, and cardiomyopathy.	22
25-year-old man	Administration of kratom powder with orange juice for 2 weeks. No other dietary supplements, alcohols, drugs (illicit, antibiotic, analgesic) were taken in this time period.	Intrahepatic cholestasis.	47

**Table 3:** Case studies on toxicities associated with kratom use similar to opioid withdrawal symptoms.

Age and sex	Dose & mode of administration	Kratom toxicity	References
44-year-old man	The subject had a history of alcohol abuse and even took oxycodone acetaminophen to treat abdominal pain. Furthermore, he took full spectrum tincture of kratom every 4–6 h with a dose of 6 dropper squeezer.	Opioid withdrawal symptoms: Abdominal cramps, sweating and diarrhea along with weight gain and severe primary hypothyroidism.	48
35-year-old man	Kratom was taken initially at a dose of 10 g/day, which later increased to 30 g/day in the form of smoothies or shakes. Along with this, sertraline and bupropion were taken to treat depression. Additionally, nicotine lozenges and caffeinated beverages were also taken on daily basis whose doses were also increases later. He had a habit of taking two glasses of wine on the weekend.	Psychological withdrawal symptoms: restlessness and irritability. Physical withdrawal symptoms: sweating, muscle spasms, headache, gastrointestinal upset, rhinorrhea and joint pain.	49
52-year-old woman	Previously, the subject had a history of opioid addiction due to the treatment of her chronic pain. Moreover, she started taking kratom powder from a quarter of a teaspoon to 1 tablespoon 4 to 6 times daily over the period of 9 months before the symptom presentation.	Opioid withdrawal symptoms: diarrhea, upset stomach, rhinorrhea, anxiety, restless legs and increased pain.	50
37-year-old woman	The subject administered kratom in the form of capsule and concentrated kratom syrup over 2 years before presentation.	Opioid withdrawal symptoms consist of severe abdominal cramps, sweats, blurred vision, nausea, vomiting, and diarrhea.	51

**Table 4:** Case studies on death associated with kratom use.

Age and gender	Dose & mode of administration	Kratom toxicity	References
26-year-old man	Unknown quantity of kratom was taken 24 h previously. He did not take any regular prescribed medication.	Death via cardiorespiratory failure and hypoxic brain damage.	52
44-year-old man	Administration of tea obtained from kratom powder for a few months before death along with taking high doses of hydromorphone on the day of his death. His regular drugs involved zopiclone, olanzapine, escitalopram, and hydromorphone (36 mg daily).	Death with high levels of creatinine and urea in vitreous humor with renal disablement. Femoral blood contained 560 ng/mL mitragynine along with 79 ng/mL hydromorphone.	53
17-year-old man	Heroin abuse and self-medication with kratom.	Death with lung congestion and edema along with a distended bladder. Whole blood analysis report revealed 0.60 mg/L mitragynine.	54
A middle-aged man	Kratom purchased online and was ingested after mixing with water.	Death with bronchopneumonia, pulmonary edema, enlarged heart, coronary atherosclerosis, and gastric ulcer. In blood, 1.06 mg/L mitragynine and 0.15 mg/L 7-hydroxymitragynine were present. Urine analysis showed 3.47 and 2.20 mg/L for mitragynine and 7-hydroxymitragynine, respectively. Also, toxicological analysis from the blood revealed the presence of zopiclone (0.043 mg/L) (lethal level: 0.6 mg/L), citalopram (0.36 mg/L) (L: 5.0 mg/L) and lamotrigine (5.4 mg/L) (L: 50 mg/L).	55
33-year-old man	He had a history of substance abuse (alcohol, marijuana, opioids, heroin, cocaine, benzodiazepines, etc.) and kratom administration.	Death with the presence of common abused substances along with mitragynine (1.9 mg/L) in his blood from the inferior vena cava.	56
27-year-old man	Administration of quetiapine and kratom.	Death with dark fluid emitted from the mouth with presence of quetiapine and mitragynine.	57

**Table 5:** Case studies on other toxicities associated with kratom use.

Age and sex	Dose & mode of administration	Kratom toxicity	References
64-year-old man	Tea made with kratom and *Datura stramonium.* His chronic pain and depression were managed with amitriptyline and oxycodone. Also, he significantly took alcohol and tobacco.	Seizure and coma associated with dry oropharynx, minimally reactive pupils, sinus tachycardia.	58
15-year-old girl	45 capsules of kratom having each dose of 500 mg for committing suicide. She did not co-administer other drugs.	Nausea, vomiting, tachycardia, xerostomia, dizziness, hypokalemia, and increased lactic acid level.	59
61-year-old man	Administration of kratom on a daily basis for the 4 months previously. He only administered rosuvastatin but no NSAIDs, antibiotic, smoking, alcohol, and illicit drugs were taken.	Unexplained persistent hyperkalemia.	60
62-year-old man	Administration of kratom. The subject was a chronic alcoholic.	Hyponatremia-associated with altered mental status and restlessness.	61
39-year-old woman	Administration of kratom from few months ago as well as 1 h of becoming affected. She didn't have any previous medical condition.	Palpitation and weakness followed by short time paralysis.	62
24-year-old man	Recreational use of alcohol and kratom (highest 600 mg of kratom daily).	Hypothermia (94.8 °F), seizure, acute respiratory acidosis, acute rhabdomyolysis, prolonged QT interval in ECG and bradycardia.	63
43-year-old man	Administration of tea made of kratom four times a day for 3.5 years; however, in the toxicity presentation day, 100 mg modafinil was co-administered with kratom. He had a history of administration and tolerance on hydromorphone.	Generalized tonic-clonic seizure with pulse 123 beats per minute.	64
20-year-old-man	Administration of tea made of kratom powder. Upon development of tolerance, the intake was every 2 h with a total daily dose up to 30 g. Also, dextroamphetamine was taken for ADHD.	Difficulties in tolerating regular dose of ADHD medicine due to tachycardia and heart palpitations.	65

In most of these case studies, the subjects were male (ranging from teenagers to old). However, an interesting case study was observed where the subject was a 1-day-old baby, who was brought to the emergency unit with symptoms similar to opioid withdrawal. The baby exhibited trouble breathing, restlessness, muscle hypertonicity, and hypoglycemia were present. During the pregnancy, the mother had taken fluoxetine and two herbal products, namely kava–kava and kratom, to manage her anxiety. Since kava–kava and fluoxetine have not been proven or documented to exhibit opioid withdrawal symptoms, it was deduced that the kratom could be the main agent responsible for manifestation of opioid withdrawal symptoms in the baby.^66^

### Other studies on kratom toxicity

Apart from the above-mentioned studies, other types of studies have also been performed to explore kratom toxicity. Among them, a cross-sectional study with 77 people, of whom 58 were regular kratom consumers and 19 healthy people (control) was performed by face-to-face interviews. The obtained kratom samples were analyzed by gas chromatography–mass spectrometry for quantification of mitragynine to correlate the consumption of mitragynine and its effects on blood parameters. The results showed no alteration in blood and clinical test parameters compared to the control group; however, the low-density lipoprotein and high-density lipoprotein cholesterol levels were found to be elevated in the kratom consumer group.^67^ Similar outcomes were observed by Singh et al.,^44^ who performed a cross-sectional study on 13 regular kratom consumers to nail down the effects of chronic kratom administration (more than 20 years). The participants underwent blood tests and other laboratory analyses. The outcomes showed no notable changes in hematological, hepatic, and renal systems of participants with no previous substance misuse history. However, chronic and heavy consumers (>3 glasses/day) showed potential cardiovascular risk by elevated lipid values along with homocysteine levels.

Apart from this, Prevete et al.^68^ conducted a study focusing on preclinical and clinical scientific literature regarding the therapeutic value of kratom. The findings of this study highlighted kratom-related toxicities and side effects, mentioning that the adulteration of kratom products sold online or toxicities in combination with other drugs could be possible reasons for these toxicities. Conversely, Abdullah & Singh^69^ highlighted in their study about the cardiotoxicity and cardiovascular system-related effects of kratom, which included tachycardia, hypertension, coronary atherosclerosis, myocardial infarction, hypertensive cardiovascular disease, left ventricular hypertrophy, cardiac arrhythmia, cardiomegaly, cardiomyopathy, focal band necrosis in the myocardium, and myocarditis.

Additionally, Yang et al.^70^ conducted a study revealed through their literature-based study on therapeutic potential of kratom on mental health-related problems along with the risks. The outcomes revealed its potential antidepressant and anxiolytic activity along with problems associated with kratom use such as dependence, cravings, and tolerance. Furthermore, a review on case studies of kratom toxicity included hypothyroidism, hypogonadism, hepatitis, acute respiratory distress syndrome, posterior reversible encephalopathy syndrome, seizure, and coma, whereas overdose can generate respiratory failure, cardiac arrest, and death.^71^ Moreover, a retrospective study was performed on 52 Kratom exposure cases of Ramathibodi Poison Center, Thailand over a 5-year period of time. The subjects were divided into two groups: poisoning cases (76.9 %) and withdrawal cases (23.1 %). Under the poisoning cases, the most common symptoms were increased heartbeat and seizure, whereas withdrawal symptoms encompassed myalgia, insomnia, fatigue, and chest discomfort. Along with this, there was a history of an infant who got withdrawal symptoms due to his/her chronic kratom abusing mother. However, no incidence of death was observed in any of these two groups.^72^

### Management of kratom toxicity

With the growing incidence of kratom toxicity, its management has become a great concern. The results of scientific studies and case reports provide insight into possible measures that could be taken to alleviate toxic symptoms and prevent further relapse. Generally, mitragynine has a high volume of distribution with very low renal clearance; hence, dialysis is not deemed beneficial after kratom overdose. Since the toxicity of kratom is mediated via stimulation of opioid receptors, opioid receptor antagonists can be used to reverse the effects. Additionally, the primary management can include the symptomatic treatment of the toxicity.^73^ For instance, Peran et al.^46^ recommended that naloxone could be used as an antidote for kratom toxicity, and for sympathetic symptoms such as hypertension, mydriasis and tachycardia, labetalol could be used. Additionally, Sheleg & Collins^48^ included a case study involving kratom toxicities similar to opioid withdrawal symptoms including hypothyroidism, where buprenorphine (a partial opioid agonist) and levothyroxine therapy were administered, and the symptoms disappeared successfully. Conversely, Agapoff & Kilaru^49^ reported an incidence of a similar pattern of kratom withdrawal symptoms including both psychological and physical symptoms. To manage the toxicities, buprenorphine/naloxone were effectively used.

Apart from these, Diep et al.^63^ presented a case, where the patient had a history of recreational use of alcohol and kratom. In this case, the patient was placed on mechanical ventilation to treat acute respiratory acidosis. Additionally, lorazepam and levetiracetam were administered to treat status epilepticus. Moreover, Ahmed et al.^74^ included a case study of kratom toxicity in the form of hypoxia and provided some suggestions or precautionary actions for handling kratom overdose toxicity. Generally, kratom overdose toxicity is similar to opioid toxicity and it poses a risk of symptoms relapse and hypoxia. Therefore, emergency department health practitioners need to be careful and should keep the patient under 24-h observation to avoid rebound risk. Moreover, Aggarwal et al.^52^ suggested that lipid emulsion therapy can be beneficial for tackling cardiorespiratory difficulties caused by kratom and in cases of calcium channel blockade, high-dose insulin might be beneficial. Furthermore, Sangani et al.^22^ suggested benzodiazepines for managing seizures and naloxone for respiratory depression. Besides these, kratom withdrawal symptoms can be managed by clonidine, naltrexone, hydroxyzine, buprenorphine, and antidepressants such as venlafaxine and sertraline.^73^

Furthermore, there have been instances where people were attracted by advertisement or other means that suggesting kratom is a safer option to treat pain and increase alertness. Since it has a natural source, people assume it to be more trustworthy. For instances, one case study revealed that a subject was attracted by an advertisement claiming that “kratom acts like an opioid without creating dependency,” which led her to take commercially available kratom powder to relieve her pain, eventually developing toxicities.^50^ Similarly, another woman was attracted by an online advertisement that kratom is a natural substance that can treat chronic pain and anxiety without creating addiction. Consequently, she started taking kratom and later, she was 16 weeks pregnant. Although she tried to discontinue kratom, she had difficulties due to the withdrawal symptoms.^75^ Therefore, mass awareness on kratom toxicities and imposing strict actions on the illegal supply of kratom can reduce the incidence of toxicity.

## Discussion

### Gap analysis

Upon reviewing various studies on kratom toxicities, it has been observed that, to date, no scientific studies have directly found incidences of lethality; however, the case studies evidenced death incidences associated with kratom use. Thus, there are substantial gaps in scientific experiments in assessing the fatalities associated with the administration of kratom-based products. The existing *in vitro* studies have only focused on the metabolizing enzymes to indirectly link kratom toxicity. Hence, more directed *in vitro* studies are required to establish kratom toxicity. Along with this, differences in the outcomes of *in vitro* and *in vivo* tests were observed. For instance, two separate studies evaluated the impacts of methanol, aqueous, and alkaloid extracts on glutathione transferase-specific activity^25^ and aminopyrine N-demethylase and UDP-glucuronosyltransferase enzyme activity^27^ via *in vitro* and *in vivo* tests. The outcomes of both studies evidenced the inhibition of enzyme activity in the *in vitro* tests, whereas an increase in enzyme activity was observed in the *in vivo* tests. Thus, more reproducible studies are required in this regard to reach a conclusion. In case of case reports, it has been observed that most incidences occurred in Western countries. By contrast, the local village people of Southeast Asia still depend on kratom leaves in their day-to-day lives; however, with the exception of a few side effects, no serious toxicities have been recorded for them. From this, an important aspect has been identified: the “kratom source.” Almost all case studies with kratom toxicities were due to administration of commercial kratom products (powder, syrup, tincture, or tea form) purchased from the internet-based market. However, there is no incidence of serious kratom toxicities from natural, fresh kratom leaves. Hence, there is a high possibility that these products were either adulterated (toxic metals or *Salmonella*) or too potent (artificially increased the concentration of alkaloids).^62^

Along with this, all of these case incidences were due to self-determined doses and some case reports evidenced that the subjects gradually increased the dose. This is notable evidence of kratom addiction and dependence. Therefore, the kratom source and lack of awareness in kratom dose determination could be the reasons for kratom toxicities. Apart from this, the lack of recording of kratom toxicity in rural people due to a lack of knowledge, awareness, and availability of proper toxicity management sections may be responsible for the fewer case studies observed on kratom toxicity. Finally, there could be some genetic or physiological differences between the inhabitants of Southeast Asia and Western countries, which has made the latter more susceptible to kratom toxicity. Therefore, more detailed and insightful scientific research should be carried out to identify genetic or physiological factors for kratom toxicity. Besides, most case reports did not report 7-hydroxymitragynine concentration in the blood/urine of the patients, which should be taken into consideration during case reporting.

### Future directions

The existing gaps provide a great baseline for further toxicological studies. Assessing the toxicity of kratom is necessary for knowing and understanding its safe consumption and potential future applications. More insightful *in vitro* and *in vivo* studies will allow to obtain reproducible results, which can be effectively correlated with case study results. Studying the contribution of genetic factors to developing kratom toxicities will help us understand this topic better. With a better understanding of its toxicity, this plant could be developed into a regulated medicinal product for pain management, opioid withdrawal, and other therapeutic uses. However, this would need thorough clinical trials to safeguard its safety and effectiveness. Establishing standardized dosages and formulations could help alleviate risks related to its use. Moreover, regulatory frameworks could be built to control its distribution and ensure quality.

Further research into the specific alkaloids in kratom, such as mitragynine and its key derivatives, namely, 7-hydroxymitragynine, paynantheine, speciociliatine, speciogynine, and corynantheidine could pave the way for the development of new drugs with targeted therapeutic effects. *In vivo* studies using different animal models should be carried out to ascertain whether the alkaloids of this plant are metabolized into more toxic compounds. Additionally, strategies should be developed to mitigate the toxic effects caused by these metabolites. Moreover, there is still room for scientific experiments to identify and remove the toxicity genes from kratom, which will further help to prepare safe medicine options for human beings.

Likewise, exploring the consumption of kratom in combination with other medications or therapies could augment its effectiveness and decrease potential side effects associated with it. Besides, more proactive reporting of toxicities will provide additional information and mass awareness will also encourage people be cautious about self-medication, especially with kratom. Utilizing and developing clear policies and legal frameworks to control the use of kratom can help balance its potential benefits with the need to safeguard and protect public health.

## Conclusion

In conclusion, the numerous scientific studies indicate that though kratom (a preparation derived from the leaves of the Southeast Asian plant *M. speciosa*) has several ethnopharmacological usages and pharmacological potential, its toxicity profile remains insufficiently understood. The gaps in our understanding persist due to the limited number of toxicological studies available which indicate potential risks, including hepatotoxicity, neurotoxicity and cardiotoxicity, specifically with high-dose or long-term of its consumption. These outcomes emphasize the requirement for more comprehensive toxicological studies to thoroughly characterize kratom's safety profile. Addressing these research gaps is imperative for informing regulatory decisions and ensuring the safe use of kratom in therapeutic contexts. Future research based on this plant should focus on long-term toxicity studies, dose-response relationships, and the identification of specific toxic compounds and metabolites within kratom as well as after its intake. Such efforts will be undoubtedly helpful in advancing the safe and effective use of kratom in medicine as well as benefiting the drug development and commercialization sectors.

## Source of funding

The authors would like to acknowledge Regal Malay Capital Berhad, Malaysia (No. SPP22-137-0137) for research support.

## Ethical approval

Not required.

## Author contributions

TB, MHA, and QUA conceived and designed the study and collected and organized data. QUA, MHA, ABMHU, AK, and SAA analyzed and interpreted the data and supervised the study. TB wrote the initial draft. All authors have critically reviewed and approved the final draft and are responsible for the content and similarity index of the manuscript.

## Conflict of interest

The authors have no conflicts of interest to declare.
